# Supplementary material for: Large gaps in monitoring urban air pollution in low- and middle- income countries associated with economic conditions and political institutions
Source: PLoS One. 2026 Jan 13;21(1):e0321358. doi: 10.1371/journal.pone.0321358 (PMC12798989; doi:10.1371/journal.pone.0321358)
Supplement: S1 Appendix — (PDF) [file pone.0321358.s001.pdf]

Supplementary Materials for

Large gaps in monitoring urban air pollution in low- and middle- income countries associated with economic conditions and political institutions

Maja Schoch, Camille Fournier De Lauriere, Thomas Bernauer

December 19, 2025

This appendix includes additional figures and tables referenced in the manuscript, including additional analysis such as a country-level Poisson regression, the effect of US-embassy monitors on other AQM, & sensitivity tests such as robustness to subsampling India and China, to different methodological decisions on results from Eq(2), and to using a different data source for income.

Contents

Supplementary material **2**

Supplementary Figures 3

S1 Table 10

S2 Table 11

S3 Table 12

S4 Table 13

Supplementary analysis and robustness checks 14

List of Figures

S1 Effects of explanatory variables for a model with only reference grade, or only air sensors, and with subsampling China and India or the full dataset. 3

S2 Interaction between democracy, pollution and GDP on AQM. 4

S3 Visualizing main results using Poisson regressions. 5

S4 Main findings are consistent when using a Poisson regression model predicting the number of monitors in a country. 6

S5 Effect Size of the interaction between democracy and pollution levels for 1000 different random subsampling of cities in China and India. 7

S6 Principal data source for each country included in the main analysis. 8

S7 Distribution of AQM for reference grade monitors and air sensors. 9

Supplementary Figures

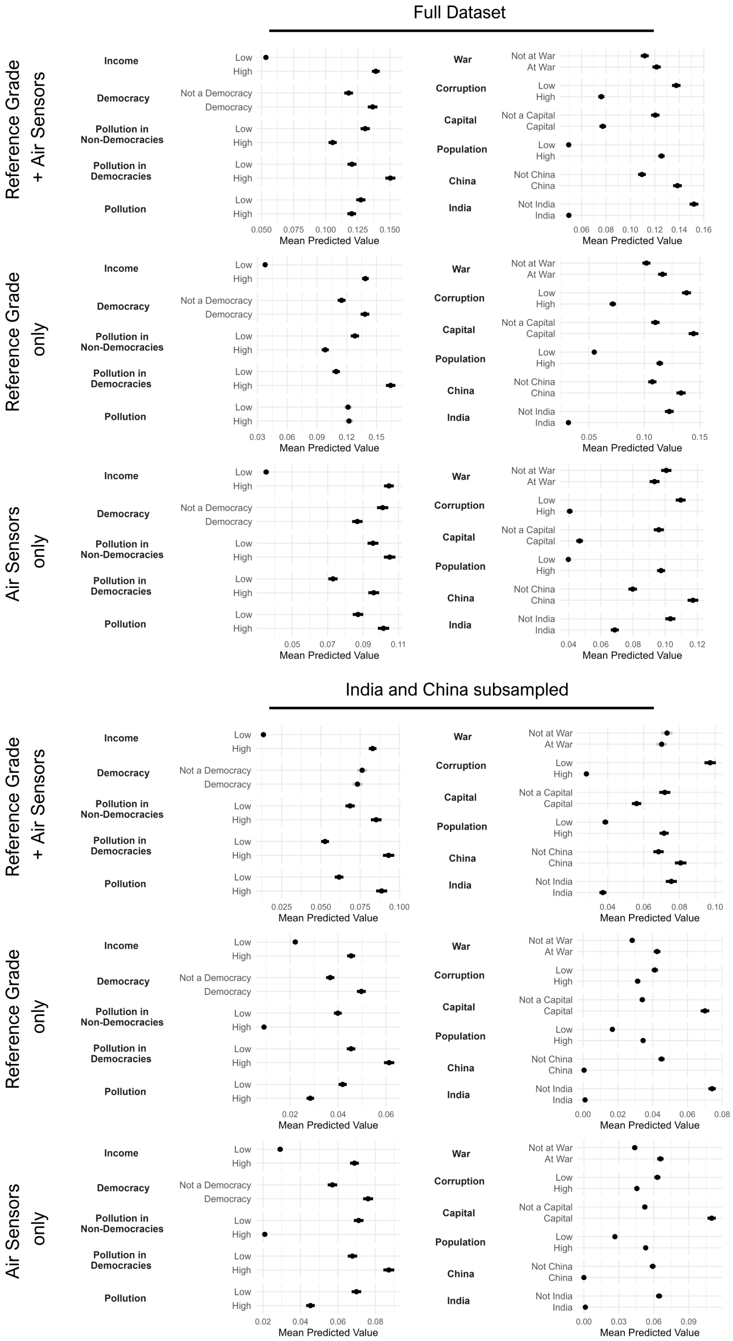

Figure S1. Effects of explanatory variables for a model with only reference grade, or only air sensors, and with subsampling China and India or the full dataset. We find that our marginal effects are very similar when using only reference grade monitors, only low-cost sensors, and when using the full dataset or applying the subsampling strategy. The interaction between democracy and pollution is robust, and minor effects such as the general effect of pollution (if the model already accounts for the interaction), as well as the impact of war, seem to be impacted by these methodological decisions.

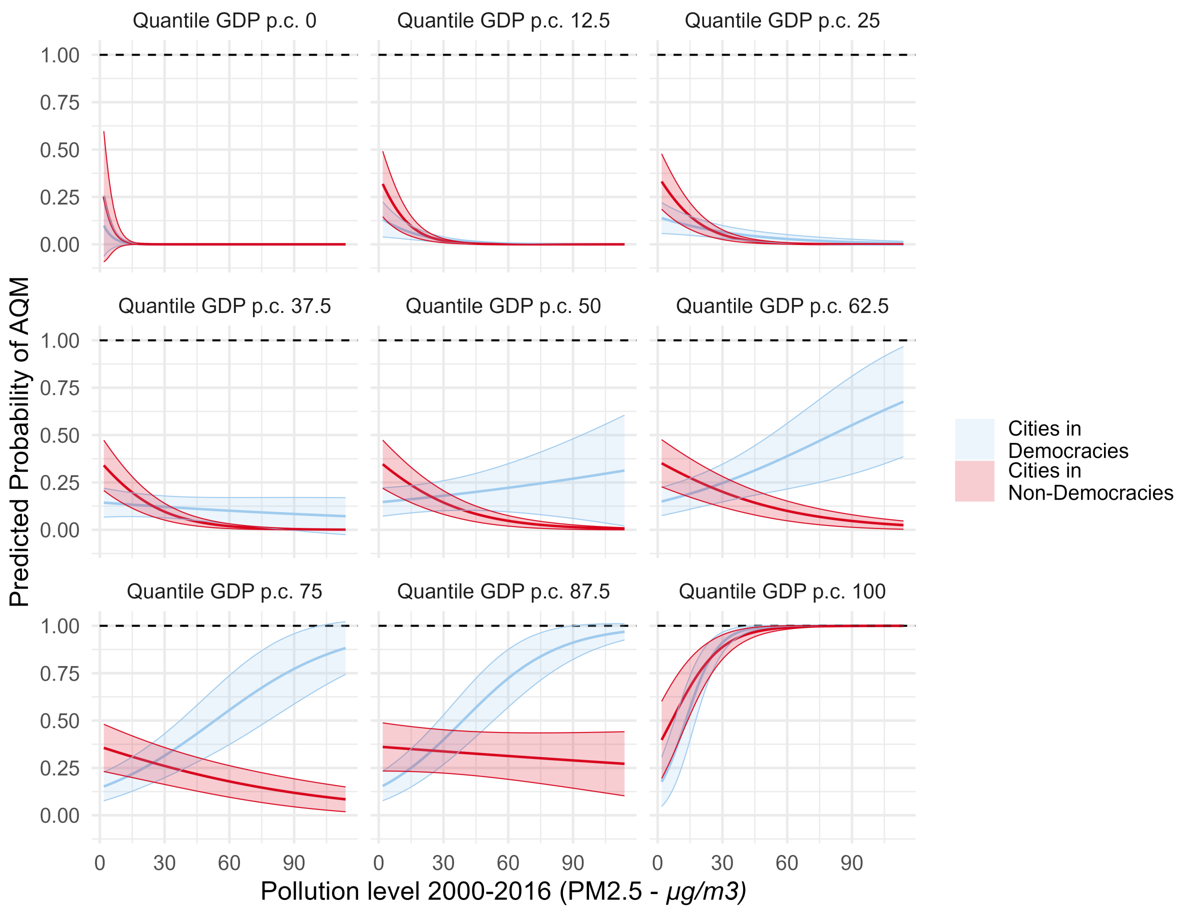

Figure S2. Interaction between democracy, pollution and GDP on AQM. The main finding in our paper — democracies are more likely to monitor cities that are more polluted — is observable in different economic contexts. Richer cities (higher GDP p.c.) are more likely to be monitored, with the poorest cities seeing little monitoring (the effect is less pronounced for non-democracies). The richest cities are very likely to monitor even if moderately polluted.

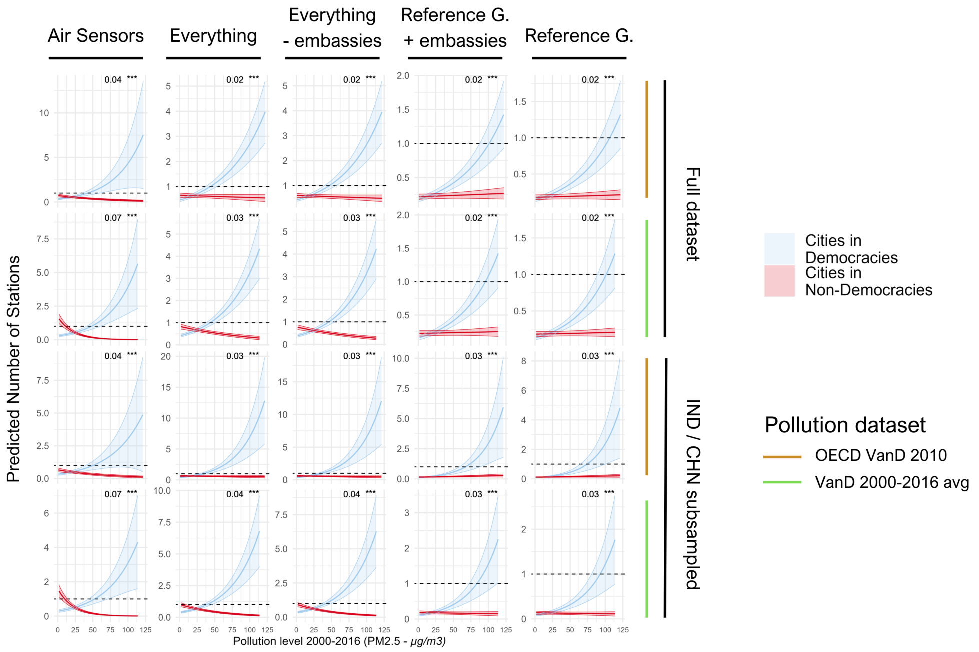

Figure S3. Visualizing main results using Poisson regressions. We test if not only the presence of AQM but also its intensity (number of monitors in each city), is higher in more polluted democracies. We find that for all combinations of inclusion of air sensors and reference grade monitors, with two different pollution datasets, and with the full and the subsampled dataset, democracies are predicted to have more AQM with increasing pollution, relative to non-democracies.

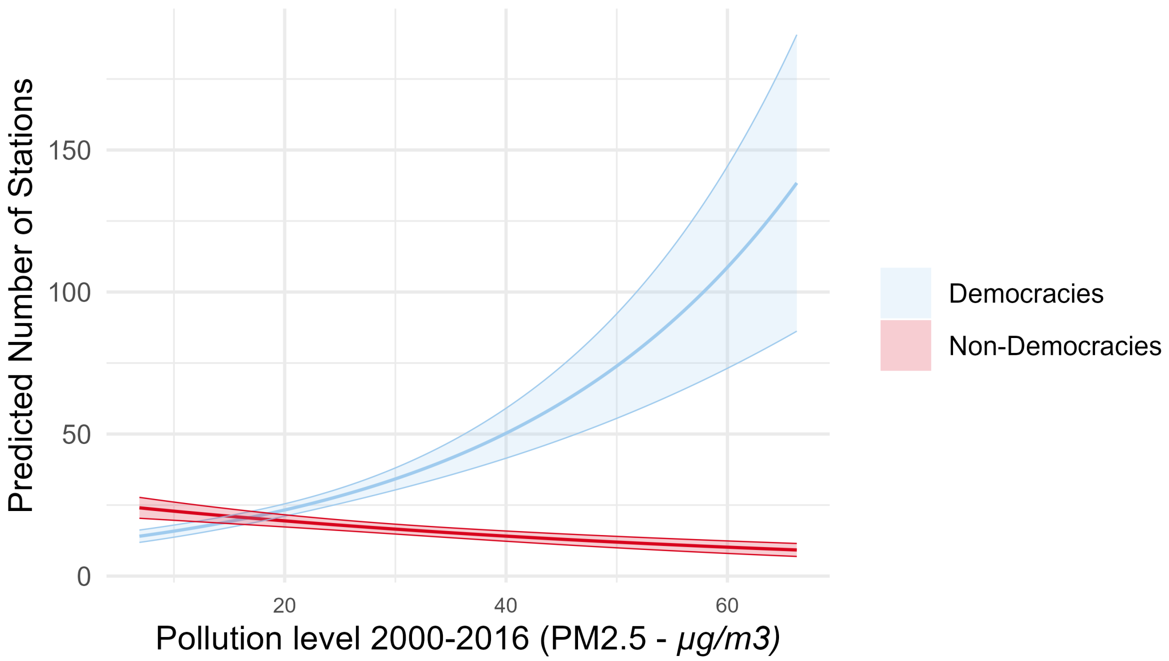

Figure S4. Main findings are consistent when using a Poisson regression model predicting the number of monitors in a country. The results show that our findings are robust at the country level, with more democratic countries having more publicly available monitoring stations with increasing pollution, while non-democracies have higher AQM if less polluted.

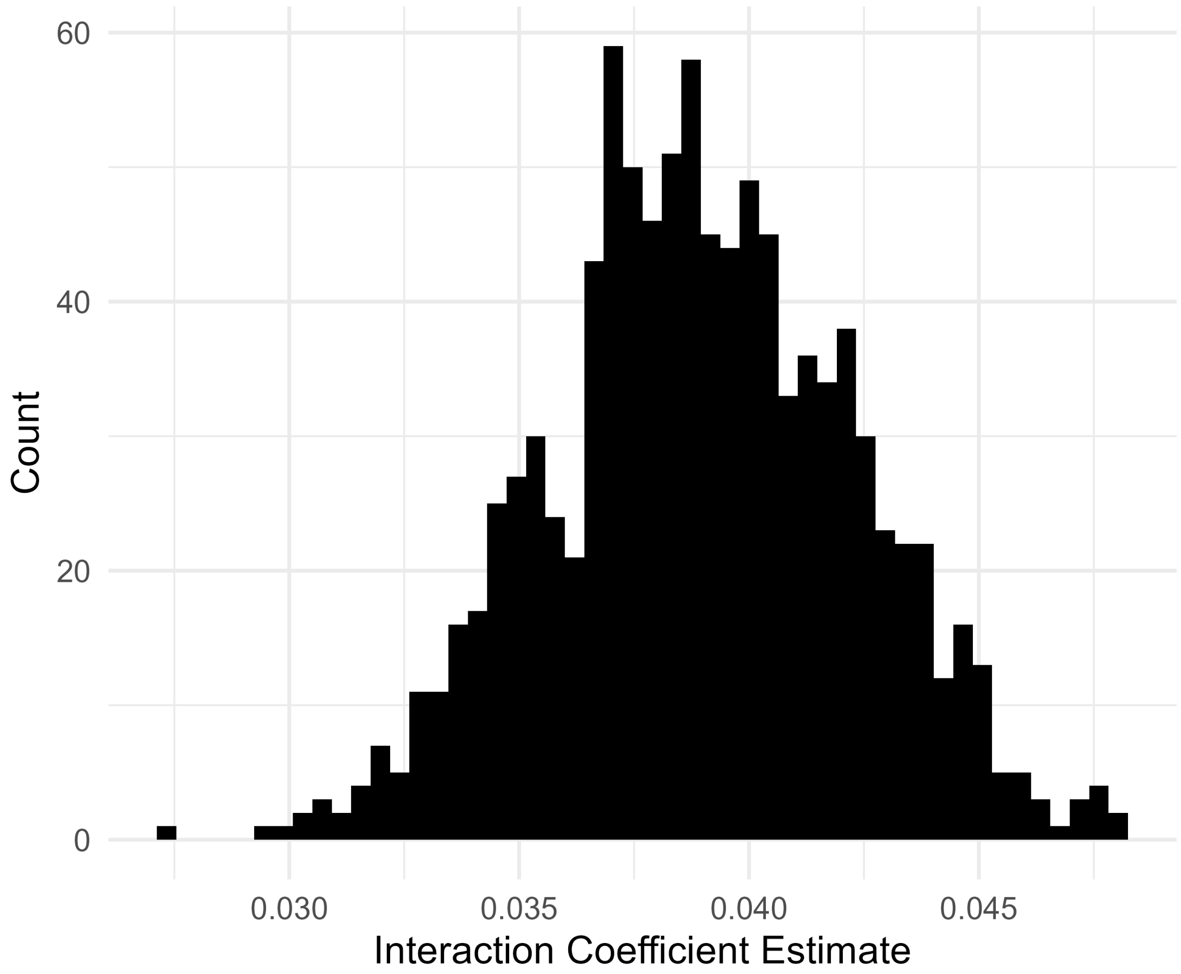

Figure S5. Effect Size of the interaction between democracy and pollution levels for 1000 different random subsampling of cities in China and India. The results are robust to the random sub-selection of cities in China and India.

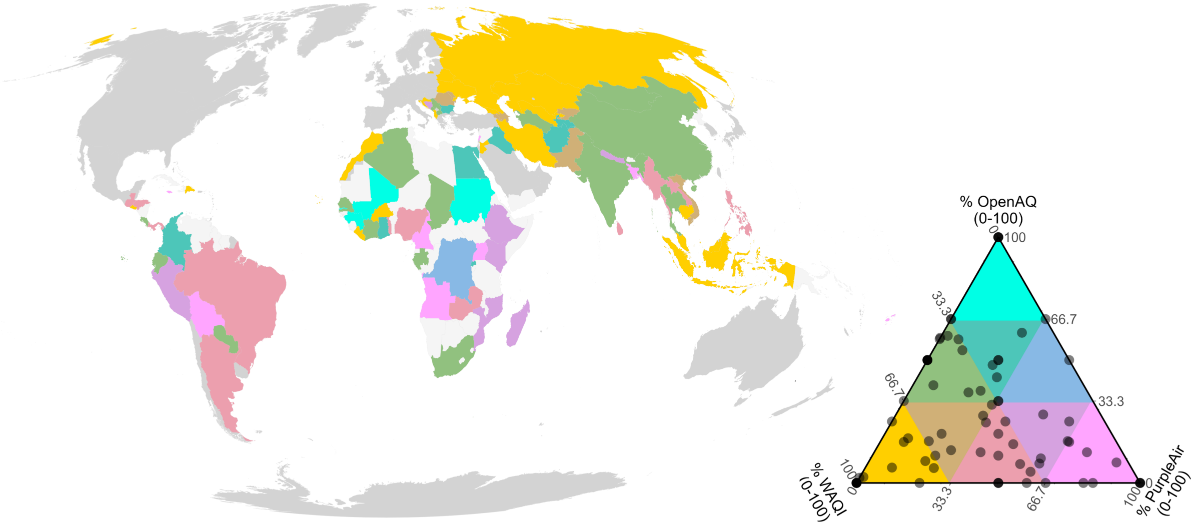

Figure S6. Principal data source for each country included in the main analysis.

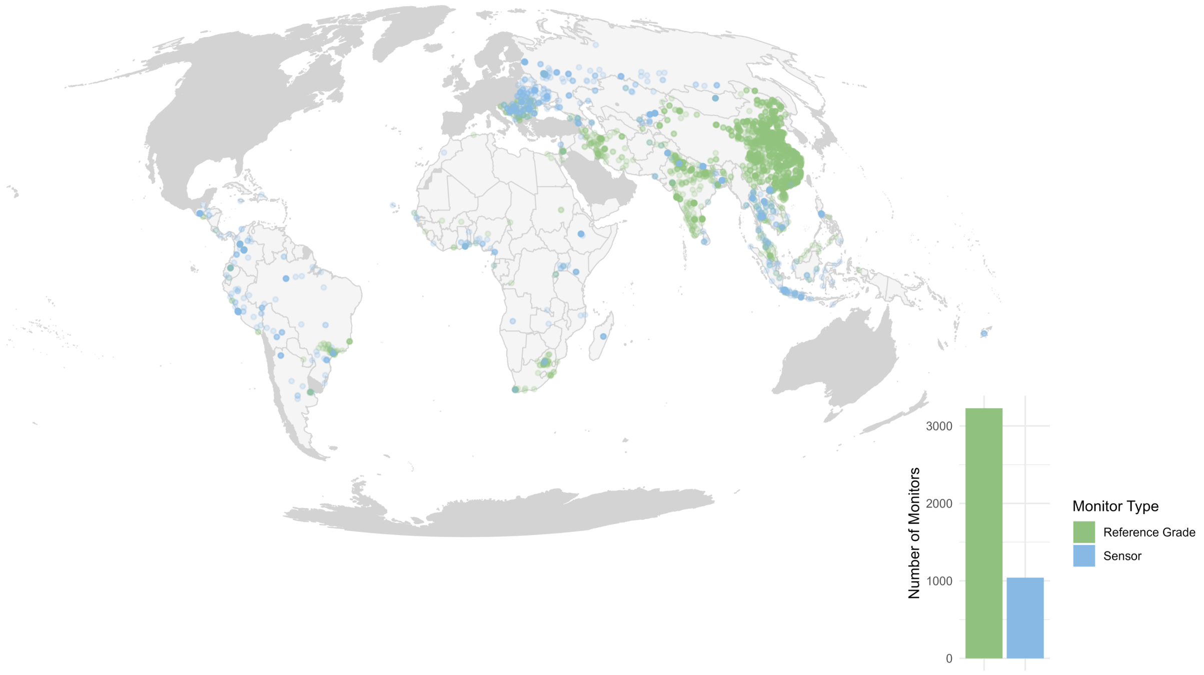

Figure S7. Distribution of AQM for reference grade monitors and air sensors.

S1 Table

Effect sizes of explanatory variables for a Poisson regression model predicting the number of monitors in a country. The results show that our findings are robust at the country level, with democratic countries having more publicly available monitoring stations with increasing pollution, while non-democracies have more AQM if less polluted.

**Variable**	**Estimate**	**Std. Error**	**t value**	**p-value**
(Intercept)	-14.876	0.539	-27.624	0.000
Log GDP per Capita	1.226	0.036	34.187	0.000
Pollution	-0.016	0.003	-5.562	0.000
Democracy	-0.915	0.124	-7.351	0.000
Country in War	0.500	0.054	9.328	0.000
Corruption	-0.019	0.003	-5.556	0.000
Log Population	0.521	0.019	27.711	0.000
Country is India	-0.232	0.154	-1.503	0.133
Country is China	1.914	0.102	18.794	0.000
Pollution × Democracy	0.055	0.005	10.901	0.000

Notes: $R^2 = 0.869$; AIC = 2878.681; $n = 121$.

S2 Table

Effect sizes of explanatory variables for a Poisson regression model predicting the number of monitors in cities that host a US-embassy monitor. We find that when controlling for other factors, such as income, population, and democracy, there are fewer reference grade monitors, and more air sensors, in capital cities that host a US embassy monitor, but we find no effect when not differentiating monitor type.

	**All Non-US Monitors**	**Reference Monitors**	**Air Sensors**
(Intercept)	-11.039*** (0.700)	-17.167*** (1.327)	-9.284*** (0.872)
Log GDP per Capita	0.842*** (0.051)	1.421*** (0.100)	0.532*** (0.060)
Pollution	-0.012*** (0.003)	0.008 (0.004)	-0.032*** (0.005)
Democracy	-0.674*** (0.131)	-0.386* (0.194)	-0.727*** (0.192)
Country in War	0.501*** (0.101)	0.591*** (0.171)	0.359** (0.128)
Corruption	-0.024*** (0.005)	-0.045*** (0.008)	-0.005 (0.006)
Log Population	0.498*** (0.039)	0.542*** (0.063)	0.478*** (0.052)
**Presence of US Embassy Monitor**	**-0.469*** (0.084)**	**-1.079*** (0.144)**	**0.039 (0.108)**
Pollution $\times$ Democracy	0.028*** (0.003)	0.023*** (0.004)	0.029*** (0.006)
AIC	1449.479	664.271	1088.351
N	119	119	119
McFadden R^2	0.560	0.701	0.344

Notes: * $p < 0.05$, ** $p < 0.01$, *** $p < 0.001$. Standard errors in parentheses.

S3 Table

Variables and relevant citations for the datasets used in the analysis.

Variable	Description	Citation / Source
Pollution	Modeled $PM_{2.5}$ (1×1 km, yearly); city mean 2000–2016 (baseline).	Van Donkelaar et al. 2021 [1]
City outline	Functional Urban Centres (1×1 km).	OECD UC dataset [2]
Population	Population in 2015 for every UC.	OECD UC dataset [2]
Income	GDP in 2015 for every UC, divided by population in 2015 (GDP per capita).	OECD UC dataset [2]
Monitoring (global)	Harmonized data from OpenAQ, WAQI, and PurpleAir.	`https://openaq.org/`; `https://waqi.info/`; `https://www2.purpleair.com/`
Democracy	V-Dem Electoral Democracy Index (country-year).	Coppedge et al. 2023 [3]
Corruption	Corruption Perceptions Index (avg. 2012–2022).	`https://www.transparency.org/en/cpi/2022`
War	UCDP/PRIO Armed Conflict Dataset; conflicts with more than 1000 battle-related deaths (2000–2022).	Davies and Pettersson 2023 [4]; Gleditsch et al. 2002 [5]
Capital	Distance to capital.	OECD UC dataset [2]

S4 Table

Effect sizes of explanatory variables for a baseline logistic regression model, predicting the presence of AQM, when using a different income level data source. We find that our results are robust to using the GDP per capita dataset from Kummu et al. 2025[6].

Variable	Estimate	Std. Error	t value	p-value
(Intercept)	-22.916	1.112	-20.602	0.000
Log GDP per Capita	1.554	0.075	20.845	0.000
Pollution	0.002	0.004	0.435	0.664
Democracy	-0.941	0.209	-4.499	0.000
Country in War	0.181	0.119	1.523	0.128
Corruption	-0.074	0.009	-7.857	0.000
City is a Capital	0.247	0.273	0.904	0.366
Log Population	0.953	0.049	19.311	0.000
City in China	0.151	0.185	0.821	0.412
City in India	-1.879	0.362	-5.199	0.000
Pollution × Democracy	0.039	0.007	5.196	0.000

$R^2 = 0.361$; AIC = 3228.068; $n = 7'429$.

Supplementary analysis and robustness checks

Robustness to a different income product: Because several cities had a GDP of 0 in the OECD UC dataset, and to test whether our main findings were robust to using a different dataset for income, we extracted GDP per capita data from the paper from Kummu et al., 2025 [6], and found that results were consistent with the OECD dataset, see S4 Table. We calculated the average GDP per capita for each UC using the spatial extent of each UC, for the year 2015.

Spatial autocorrelation: We tested whether monitors were more likely to be placed when other monitors were placed in neighboring cities. Essentially, we account for city- and country-level variations in our regression, but regional scale policies might make neighbor cities more likely to host AQM. While answering this question requires a deep analysis that is outside the scope of that paper, we calculate spatial autocorrelation (Moran's I) of the residual variation from our logistic regression for a radius of 50, 100, 500 and 1000km around each city. We find that this coefficient is positively statistically significant, with the largest effect for a 100km radius around cities (Moran's I = 0.26, $p < 0.01$). This means that cities are more likely to host a monitor if other cities in a 100km radius also host one. This suggests that regional governance may drive monitor placement, which we do not explicitly quantify in this research.

References

[1] Aaron van Donkelaar, Melanie S. Hammer, Liam Bindle, Michael Brauer, Jeffery R. Brook, Michael J. Garay, N. Christina Hsu, Olga V. Kalashnikova, Ralph A. Kahn, Colin Lee, Robert C. Levy, Alexei Lyapustin, Andrew M. Sayer, and Randall V. Martin. Monthly Global Estimates of Fine Particulate Matter and Their Uncertainty. *Environmental Science & Technology*, 55(22):15287–15300, November 2021. ISSN 0013-936X. doi: 10.1021/acs.est.1c05309. URL `https://doi.org/10.1021/acs.est.1c05309`. Publisher: American Chemical Society.

[2] Marcello Schiavina, Michele Melchiorri, and Martino Pesaresi. GHS-SMOD R2023A - GHS settlement layers, application of the Degree of Urbanisation methodology (stage I) to GHS-POP R2023A and GHS-BUILT-S R2023A, multitemporal (1975-2030). May 2023. doi: 10.2905/A0DF7A6F-49DE-46EA-9BDE-563437A6E2BA. URL `http://data.europa.eu/89h/a0df7a6f-49de-46ea-9bde-563437a6e2ba`. Publisher: European Commission, Joint Research Centre (JRC).

[3] Michael Coppedge, John Gerring, Carl Henrik Knutsen, Staffan I. Lindberg, Jan Teorell, David Altman, Michael Bernhard, Agnes Cornell, M. Steven Fish, Lisa Gastaldi, Haakon Gjerløw, Adam Glynn, Sandra Grahn, Allen Hicken, Katrin Kinzelbach, Kyle L. Marquardt, Kelly McMann, Valeriya Mechkova, Anja Neundorf, Pamela Paxton, Daniel Pemstein, Oskar Rydén, Johannes von Römer, Brigitte Seim, Rachel Sigman, Svend-Erik Skaaning, Jeffrey Staton, Aksel Sundström, Eitan Tzelgov, Yi-ting Wang, Tore Wig, Daniel Ziblatt, Ana Good God, Joshua Krusell, Juraj Medzihorsky, Natalia Natsika, Josefine Pernes, and Wilson. ”V-Dem [Country-Year/Country-Date] Dataset v13” Varieties of Democracy (V-Dem) Project, 2023.

[4] Shawn Davies, Therese Pettersson, and Magnus Öberg. Organized violence 1989-2022 and the return of conflicts between states? *Journal of Peace Research*, Forthcoming, 2023.

[5] Nils Petter Gleditsch, Peter Wallensteen, Mikael Eriksson, Margareta Sollenberg, and Håvard Strand. Armed Conflict 1946-2001: A New Dataset. *Journal of Peace Research*, 39(5), 2002.

[6] Matti Kummu, Maria Kosonen, and Sina Masoumzadeh Sayyar. Downscaled gridded global dataset for gross domestic product (GDP) per capita PPP over 1990–2022. *Scientific Data*, 12(1):178, January 2025. ISSN 2052-4463. doi: 10.1038/s41597-025-04487-x. URL `https://www.nature.com/articles/s41597-025-04487-x`. Publisher: Nature Publishing Group.
