# Supplementary material for: Large gaps in monitoring urban air pollution in low- and middle- income countries associated with economic conditions and political institutions
Source: PLoS One. 2026 Jan 13;21(1):e0321358. doi: 10.1371/journal.pone.0321358 (PMC12798989; doi:10.1371/journal.pone.0321358)
Supplement: Attachment — Submitted filename: revisionMemoPLOS.pdf [file pone.0321358.s003.pdf]

PONE-D-25-11715 Response Memo
"Large Gaps in Monitoring Urban Air Pollution in Low- and Middle- Income Countries Associated with Economic Conditions and Political Institutions"

Dear editorial team of *PLOS ONE*, dear reviewers,

Many thanks for all your very helpful comments and suggestions. We have thoroughly revised the manuscript in response to these comments and suggestions, and we are pleased to herewith resubmit the revised manuscript "Large Gaps in Monitoring Urban Air Pollution in Low- and Middle- Income Countries Associated with Economic Conditions and Political Institutions" (PONE-D-25-11715).

In this memo, we respond point-by-point to all reviewer and editor comments, and also submit one version of the revised manuscript with track changes, along the manuscript without track changes.

We believe that the paper has benefited very much from your comments and suggestions, and the resulting revision. We greatly appreciate the opportunity to revise the manuscript for consideration at *PLOS ONE* and look forward to your response. It goes without saying that we are very much willing to undertake any further revisions you might deem necessary in order to get the paper published in *PLOS ONE* .

In addition to the point-by-point response, please note that we have slightly edited all figures from the manuscript because of a small update to the AQM dataset. Some locations were wrongfully filtered out and others were duplicated. We carefully checked whether this led to significant changes in our interpretation of the findings, and adjusted when necessary, which only concerned the mentioning of descriptives in the results section.
We also made sure the countries of interest for our study were referred to, and defined consistently throughout the document, and edited title, abstract, and manuscript to refer to low- and middle- income countries (LMIC). Also, because Figure 6 and (former) Supplementary Figure 4 covered all combinations of the data displayed in (former) Supplementary Figure 3, we deleted (former) Supplementary Figure 3.

The authors

Reviewer #1:

The manuscript explores the current status of air quality monitoring (AQM) in urban areas of the Global South (low to middle income countries), its distributional disparities, and its association with economic conditions and political systems. Based on a geocoded dataset of more than 10,000 urban areas, the authors reveal large gaps in the distribution of AQM in the Global South and explore key factors influencing AQM activities. There are a number of issues that need to be revised before the article is published. My comments are below:

1. The article finds that cities in democracies are more inclined to increase AQM activity at high pollution levels, but this correlation does not necessarily imply causation. For example, democracies may themselves have a stronger economic base and public awareness, and these factors may have contributed to both the development of AQM and the establishment of democratic institutions. The article fails to adequately control for these potential confounding factors, thereby weakening the reliability of causal inferences.

Response #1.1:

Thank you very much for flagging this limitation of the paper. We fully agree that identifying causal effects of democracy is impossible because democracy cannot be manipulated experimentally or quasi-experimentally in any meaningful study design. We have revisited the paper and adjusted its wording to make it very clear that we talk about association or correlation, and not causation. Regarding the issue of confounders/control variables, we have probably not made it clear enough that our models do control for various confounding factors. For instance, we include income levels alongside democracy scores in every model, and also look at the combined (interactive) effect of income and democracy. To our knowledge, there is no reliable data for public awareness at the city or country scale that we could use to control for this potential confounder, given that we compare around 10000 cities. However, as we include pollution levels, income levels, and democracy levels in all models we believe that these factors do at least to some extent, indirectly, proxy for awareness levels, because many micro-level studies show that individuals with higher incomes living in more polluted settings with higher levels of democracy (and thus also higher levels of freedom of science and the press) tend to be more aware of environmental problems. One (albeit rather indirect) proxy for awareness we considered is The Worldwide Governance Indicators' Voice and Accountability. But this measure is highly correlated with our democracy score (Pearson's R = 0.956*), meaning that it makes no sense to include it in our models. In other words, we believe that our models now include most of the confounders that, from a theoretical perspective, might also affect monitoring behaviour, and for which we can obtain data of reasonable quality and scope. However, we are very open to including further confounders the reviewer might have in mind, and for which there is data.*

2. The article points out that non-democratic countries perform poorly on AQM, but fails to explore how monitoring capacity in these countries could be improved through external pressures or incentives. For example, can the international community promote AQM development in non-democratic countries through technical assistance, financial support, or policy advocacy?

Response #1.2:

We fully agree that this is a very interesting and important question. One might hypothesize that in lower income contexts AQM capacity can be increased mostly via country-external support, and that such support is more likely to materialize and make a difference in more democratic settings. Our empirical data analysis part

as such cannot provide an answer to this question as this would require city-level data on how much foreign assistance for AQM is flowing into which city and what the effects of it are. To flag this important issue, we added the following text to the discussion section: "Country-external governmental or non-governmental actors could catalyze more AQM via technical assistance (e.g. calibration, open-data systems), co-financing of reference backbones and low-cost densification of monitoring networks. They could also create more pressure on policy-makers to make monitoring results public and allow for independent audits. One interesting hypothesis to study in further research could be whether cities in more democratic contexts are more likely to receive such assistance and make more efficient and effective use of it."

3. The need for the study could be further emphasized by adding to the background introduction some discussion of the trend towards rapid urbanization in LMIC and how this trend is exacerbating the air pollution problem.

Response #1.3:

Thank you for pointing this out. We have added this text to the introduction: "[...] These problems are amplified by the fact that urban air pollution coincides and interacts with climate change and other ecological stressors, which implies that substantial synergies and co-benefits could be achieved from pursuing both clean air and climate mitigation measures (Cao et al. 2025, Dong et al. 2022; Vandyck et al. 2018)."

4. The introduction describes air pollution and the serious threat to public health and the environment. However, the content is not comprehensive enough and should include aspects of climate change, urban thermal degradation, urban sprawl, and ecological stress. It is recommended to refer to the following literature to enhance the comprehensiveness. -The Dynamic Effects of Ecosystem Services Supply and Demand on Air Quality: A Case Study of the Yellow River Basin, China; Investigating the attribution of urban thermal environment changes under background climate and anthropogenic exploitation scenarios; Designing green walls to Designing green walls to mitigate fine particulate pollution in an idealized urban environment; Can green finance improve China's haze pollution reduction? The role of energy efficiency; Building a climate-adaptive city: A study on the optimization of thermal vulnerability; Spatial and Temporal Heterogeneity of Human- Air-Ground Coupling Relationships. Air-Ground Coupling Relationships at Fine Scale; Combined effects of urban forests on land surface temperature and PM2.5 pollution in the winter and summer? summer

Response #1.4:

Thanks for pointing this out. If the editor allows (in view of editorial guidelines, including limits on references), we would like to add more references to emphasize how air pollution also relates to other urban environmental degradation issues. See also Response 1.3

5. In the discussion section, it is suggested to further explore why non-democratic countries tend to carry out AQM even when the pollution level is low, which may be related to the political motivation or international pressure of these countries, and adding this discussion can enhance the depth of the study.

Response #1.5:

This comment is very similar to the next one, so we have addressed both points under response #1.6.

6 The article mentions that "non-democratic countries may choose to monitor in less polluted areas", but does not explore in depth the potential reasons for this choice. It is suggested that additional hypotheses or literature support be added to explain this phenomenon.

Response #1.6:

Thanks a lot for noting this. We also found this result very interesting. At this stage, the finding must remain an empirically observed pattern. Systematically identifying the potential causes for this would probably require qualitative interviews with key officials in (hundreds of) such cities, which is far beyond the scope of our paper. However, one candidate explanation could be that policy-makers in less democratic settings have more leeway to place monitoring devices in (relatively) less polluted locations in order to make the city's pollution situation look better than it actually is. In the discussion section, we flag this hypothesis as worthy of further research. In any event, differences between reference-grade and low-cost sensor prevalence we observe suggest that in non-democracies, governments set up one reference-grade station and commonly leave it at that, whereas low-cost sensors proliferate more in highly polluted areas of the more democratic LMIC.

We revised the discussion section as follows:

"The observation that non-democracies are more likely to monitor in lower pollution areas suggests that non-democracies' monitoring locations may be biased by political considerations. While non-democracies may establish monitors in cleaner places in response to international pressures, democracies may prioritize highly polluted cities and establish more extensive monitoring networks there due to electoral incentives to provide public environmental goods. It is worth noting, however, that although environmental agencies in democracies generally operate with greater autonomy, instances of strategic misreporting have been documented in both the US and in China (Grainger and Schreiber 2019; Mu et al. 2024)."

In essence, while the research presented here can identify general patterns in the democracy-AQM relationship, the causal mechanisms driving particular patterns require further research. "

7. When discussing future research directions, it is recommended to be more specific and suggest actionable research proposals, such as how to improve monitoring networks using low-cost sensors, or how to assess the fairness of AQM in different socio-economic contexts.

Response #1.7:

Absolutely. We fully agree and added more specific suggestions on how different research designs could help answer important questions about AQM. For instance, we added the following text: "This would require finer grained data than what was used in this paper to capture within-city variations, and could be coupled with qualitative interviews to describe the process of AQM placement. [...] Experimental study designs, such as RCTs, randomizing the placement of low-cost monitors could help us understand the impact that more localized air quality information may have on people's awareness, concern, behavioral changes to reduce pollution exposure, and policy-preferences and demands."

We also added a citation (discussion) pointing to a recent review, discussing avenues for low-cost sensor networks (Bagkis et al. 2025). In addition, we also added various suggestions for further research as noted in the responses to the comments further above.

Reviewer #2:

This article explores the current state and influencing factors of air quality monitoring (AQM) in cities of the Global South. The research perspective is novel and offers interesting insights for improving air quality governance. My suggestions are as follows:

1.Data-related aspects, How can OpenAQ, WAQI, and PurpleAir implement strict controls over data quality, such as checking data consistency, completeness, and accuracy? How can more detailed analysis and processing of outliers be conducted? In addition to PM2.5, could other pollutant indicators, such as sulfur dioxide (SO_2) and nitrogen oxides (NO_x), be included to provide a more comprehensive assessment of air quality?

Response #2.1:

Thank you for raising this important issue. Each data provider on whom we rely aims at removing inaccurate measurements and identifying consistently defect monitors. OpenAQ's recent API updates add the option of downloading the number of valid measurements compared to the expected number of measurements for such a sensor (https://docs.openaq.org/resources/measurements#summary). WAQI uses data from nearby instruments to calibrate and identify outlier measurements and filter them out (discussion with an engineer from WAQI, and their website states https://aqicn.org/contact/ "a set of real-time AI algorithms are used to detect abnormal data conditions (sparks, low reporting, etc.) and automatically 'disable' data reported from defective stations.". Additional analysis of air pollution measurements' accuracy would require significant data processing from all these sources, which is far beyond the scope of our present research. Thus, we rely on the provider's quality control for the identification of defect monitors. As an additional precautionary measure, we only include monitors that were active for more than 30 days in OpenAQ or PurpleAir, or that were active for at least 3 weeks in the month of April 2024 for WAQI. We added a clarification of this to the Methods section, which mirrors the reviewer's comment here: "Data providers like WAQI and OpenAQ use data validation algorithms to identify outliers and abnormal measurements. As an additional step, we exclude monitors that were reporting for less than 30 days, and those that reported for less than 2 weeks on the WAQI API in the month of April 2024." Please note, however, that the purpose of our study is not to explain pollution levels (for which we would have to be far more careful about the quality of reported pollution levels), but simply to explain whether cities do or don't have any observable AQM. And even with our rather crude definition of this outcome variable, we can detect such activity in only around 10% of all cities in our sample.

Additionally we added the following sentence to the discussion: "[...] Future work could also examine how platforms like OpenAQ, WAQI, and PurpleAir are improving the transparency and robustness of their data quality practices, including outlier detection and handling.

2.Analytical methods, Beyond binary logistic regression and Poisson regression, are there other models that could be considered? Could the interaction between democratic systems and pollution levels be analyzed more deeply, such as exploring how this interaction varies under different levels of economic development?

Response #2.2:

Thank you very much for the suggestion to consider alternative statistical estimation strategies. We used logistic and Poisson regressions as they allow us to model both the presence of a monitor, and the number of monitors in each city, or country. These are the state-of-the-art estimation strategies for the kinds of data we have. We also considered a less conventional, machine-learning based approach (gradient boosting), to train a model to predict whether a city should have a monitor. This approach is less well established and in some ways has a black-box flavor. Unfortunately, the performance of that machine-learning model was very weak, likely due to the imbalance of the number of cities that have a monitor, against those that do not. Consequently, we decided to not rely on interpretations from SHAP-values in the absence of a decent model performance, to avoid misinterpretations. Thus, we ended up not using the machine-learning approach and tested the robustness of both city level logistic and Poisson regression results mainly based on subsets of our data and differing model specifications. Our main results turn out to be robust, also when using a Poisson regression at the country-level (see Supplementary Table 1).

Additionally, in Supplementary Figure 2 we provide a visualisation of how democracy and pollution levels interact at different levels of economic development. We find that low levels of economic development are very unlikely to be associated with any monitoring, regardless of the political system, and very high levels of pollution make no difference between political systems. Democracies seem to be more responsive to increasing pollution levels at median and high levels of economic development.

3.Spatial considerations, Could spatial autocorrelation be taken into account? Spatial statistical methods, such as spatial autoregressive models, could be used to analyze the spatial autocorrelation of AQM activities and explain spatial distribution patterns.

Response #2.3:

Thank you for this valuable comment. We consider the presence or absence of a monitor to reflect a political decision that may be driven by local and/or national actors. While regional actors may be motivated in monitoring cities in their area of jurisdiction, national actors may favor a proliferation of monitors across the country to identify hotspots of pollution exposure. While this is an interesting question, it is very challenging to deal with from an empirical modeling viewpoint and is beyond the scope of our paper. A measure of spatial autocorrelation on the residuals of the logistic regression reveals that spatial autocorrelation is significant for cities when considering a radius of 50, 100, 500, and 1000km, and is largest (Moran's I = 0.26) for 100km, suggesting that cities in the same region have the highest chance of displaying the same outcome (hosting a monitor, or not), after correcting for the covariates as described in Eq(1).

In the discussion section, we now flag this as an interesting opportunity for further research on the spread or aggregation of monitors within countries or administrative units. "We analyze city and country level determinants, but a superficial analysis of spatial autocorrelation suggests that cities within about 100 km of each other tend to share similar likelihoods of having monitoring stations, highlighting the role of regional governance."

4. Case studies, Could cities with particularly prominent or lagging AQM activities be selected for in-depth case analysis to better understand the influencing factors and mechanisms of AQM activities?

Response #2.4:

This is a great idea, though it is beyond the scope of this paper. In fact, we are planning to engage in such case studies in the coming months. The limitations of this approach, however, are that it is very time and resource-intensive and must be limited to a few cities, whereas the aim in this paper is to find out whether there are any generalizable patterns when looking at all cities of LMIC. We have already conducted a series of interviews and have summarized the findings in a paper that is currently in RandR. We will build on this and conduct additional interviews in the months ahead. Here is the title and abstract reflecting our work on this as of now:

Contextual Factors Shape Stakeholder Dynamics in Air Pollution Control - Insights from Six Majority World Cities

Abstract

Air pollution is a major global health hazard, causing millions of premature deaths annually, with the worst impacts in Majority World cities. In these contexts, diverse local conditions shape stakeholders' ability to influence government mitigation efforts. Drawing on 63 semi-structured interviews with 72 air pollution experts and stakeholders in six highly polluted cities (Accra, Kampala, Johannesburg, Jakarta, Dhaka, Delhi), we examine how economic development, democratic governance, and data availability structure the influence of key actors—citizens, local advocates, economic elites, and country-external agencies—on government mitigation efforts. We find that governments remain primarily responsive to economic elites across levels of economic development. In the lowest-income case (Dhaka), external actors play a more pronounced role. Democratic governance enables broader stakeholder engagement, especially among citizens and local advocates. Data availability expands opportunities for influence but also provokes governmental counter-strategies. These dynamics underscore opportunities and constraints for stakeholder engagement in pollution governance.

5.Network management, How can AQM networks be constructed and effectively managed to enhance monitoring efficiency and data quality?

Response #2.5:

Thank you for this comment regarding the implementation of AQM networks. While our research presented here does not provide technical solutions for designing AQM networks, but rather describes and explains observable monitoring activity, we added a reference to relevant papers in the discussion section, for instance, Bagkis et al. 2025 and Anggraini et al. 2024

6.Policy recommendations, Could concrete improvement suggestions be proposed? Can implementing policies and mitigation measures effectively improve air quality? For example, references could be made to studies on ventilation pathways (10.1016/j.buildenv.2018.09.010, 10.1016/j.scs.2019.101487) and green infrastructure (10.1016/j.scitotenv.2022.155307)?

Response #2.6:

Thank you for pointing this out. While our study does not focus on mitigation measures more broadly and does not assess whether more monitoring is likely to lead to reduced air pollution, we agree that these two issues are very important. We make it more explicit in the introduction what the paper does and does not cover, and add references to the aspects we do not cover. For instance, we refer to the emerging literature on whether more

monitoring can contribute to lowering air pollution and also refer to policy measures that are being used to reduce air pollution in LMIC. As such, we added this text to the discussion:

"While action to reduce air pollution exposure is urgent, and policies may not always necessitate a large network of AQM (Smith et al. 2024), more information can help create pollution alert systems (D'Antoni et al. 2019; Mampitiya et al. 2023), and help raise public awareness, raising support for policies aimed at tackling air pollution."

Additionally, we added a citation about environment and air pollution mitigation co-benefits "Beyond direct health harms, urban air pollution interacts with climate and ecological stressors, presenting direct co-benefits for air and climate (Cao et al. 2025, Dong et al. 2022; Vandyck et al. 2018) - see also response 1.4.

Reviewer #3:
Rewrite abstract focusing on methodology and core results.

Response #3.1:

We have revised the abstract to make the methodology and key results clearer.

State research objectives explicitly and concisely.

Define research gap clearly in a separate paragraph (Lines 84-91). Right now, it reads like a general essay.

The novelty is hidden. Explicitly claim your contribution

Response #3.2:

We agree and have revised the abstract and introduction. See also previous comment.

Avoid long-winded methodology description. Use tables to summarize datasets and variables

Exclude too much justification like "we are aware..." (Line 217), this belongs to discussion, not methods.

Response #3.3:

Thank you for this comment. We revised the methods part accordingly, and more description was added as supplementary material. We also added a table to describe every variable and its data source, see Supplementary Table 3.

Over-dependence on p-values from permutation test without effect size is weak. Show effect size more prominently.

Response #3.4:

Thank you for pointing this out. We added the mean difference between each group along with the p-values in the result section.

Discuss limitations more critically

Compare your findings directly with past literature, mention what aligns or contradicts with others.

In conclusion, Practical implications are shallow.

Response #3.5:

Thank you for this comment. We have revised the discussion section accordingly to address limitations of our research more explicitly and note how our findings relate to the (very few) other studies on the subject. We have also revisited the practical implications.

Read these studies to further enrich your study. (https://doi.org/10.1016/j.hazadv.2023.100395) Provides machine learning applications on air quality prediction in a lower-income country. Useful to highlight methodological advancements in data-scarce regions like the Global South. (https://doi.org/10.3390/environments10080141) Discusses urban air pollution modeling in Sri Lanka using ML. Strengthens the argument on challenges of urban AQM in lower-income cities. (https://doi.org/10.1016/j.rineng.2024.102831) Demonstrates SHAP-based explainable AI in environmental monitoring. Can support the significance of public trust in AQM data transparency. (https://doi.org/10.1007/s11356-025-36292-9) Discusses urban environmental monitoring using AI in developing settings. Adds depth to the environmental policy-tech interface discussion.

Response #3.6

Thank you for pointing us to these papers. We discuss the use of machine-learning (and explainable AI) approaches in Response 2.2. Eventually, we decided to rely on more conventional methods (logistic, and poisson regression) due to poor performance of XGBoost models on our dataset. We added a sentence in the last paragraph of our discussion, mentioning the role of AQM in reducing air pollution exposure, and highlighting how information can help design alert systems for high pollution events.

"While action to reduce air pollution exposure is urgent, and policies may not always necessitate a large network of AQM (Smith et al. 2024), more information can help create pollution alert systems (D'Antoni et al. 2019; Mampitiya et al. 2023), and help raise public awareness, raising support for policies aimed at tackling air pollution.

Reviewer #4:

In this paper, the authors propose an innovative approach to monitoring urban air pollution in the Global South. As a result, they have constructed a novel geocoded dataset on air quality monitoring (AQM) in more than ten thousand urban areas in low- to middle-income countries. The authors used the perm package and a Monte Carlo approximation, with 1,000 iterations, to investigate the factors that influence monitoring behavior. To test the hypotheses, the initial strategy involved bivariate analysis. The authors examine variation in air quality management (AQM) practices across cities in the developing world and explore the potential factors that contribute to this variation. The study reveals a significant disparity in AQM implementation in most urban areas in the developing countries. Economic and political conditions are identified as the primary drivers of this discrepancy. While income levels are likely to have a positive effect on AQM standards, the impact of increased air pollution levels may depend on the level of democratic governance. Thus, the article is well written and read with interest. The strength of this research lies in its

emphasis on exploring complex and unobvious relationships between democracy, environmental pollution, and gross domestic product (GDP). However, this study does have a number of limitations, the most significant of which is the weak validity of the proposed methodology due to the limited use of ecological and eco-mathematical modelling in the study. The following are recommendations for enhancing the article:

1. The literature review could be enhanced. In particular, it would be beneficial to include research on agent-based modelling of ecological and economic systems within the scope of the review. Within this context, the following significant works should be mentioned and cited:

[1] Akopov A.S., Beklaryan L.A., Saghatelyan A.K. Agent-based modelling for ecological economics: A case study of the Republic of Armenia. Ecological Modelling, Vol. 346, 2017, pp. 99-118,

https://doi.org/10.1016/j.ecolmodel.2016.11.012.

[2] Achilleas Karakoltzidis, Anna Agalliadou, Marianthi Kermenidou, Fotini Nikiforou, Anthoula Chatzimpaloglou, Eleni Feleki, Spyros Karakitsios, Alberto Gotti, Dimosthenis A. Sarigiannis, Agent-based modelling: A stochastic approach to assessing personal exposure to environmental pollutants – Insights from the URBANOME project. Science of The Total Environment, Vol. 967, 2025, https://doi.org/10.1016/j.scitotenv.2025.178804.

Response #4.1:

Thank you very much for this valuable comment. We have added these references to the relevant sections of the paper. As to the validity of the methods and the robustness of the findings, we have made sure to more address the limitations of our study and clarify methodological decisions to reduce ambiguity. Based on specific comments from referees, we added several robustness checks, see Supplementary Information. We answer specific comments in more detail below.

2. In the introduction, it would be beneficial to more clearly articulate the purpose of the research and its primary contribution. At present, there is no clear delineation of the scientific innovation of the proposed methodology. Is it the identification of new correlations, the enhancement of the accuracy of monitoring urban air pollution in the Global South, or some other aspect? More precise definitions are required. As mentioned above, it is essential to enhance the component related to the utilization of environmental, eco-economic, and eco-mathematical modelling tools.

Response #4.2:

Thank you very much for noting this. We have revised the abstract and introduction accordingly. See also our response to response 3.2.

3. The section on data and methods should be enhanced. It is necessary to add a subsection on "Ecological Modeling" or "Data Modeling". The most significant of which is the weak validity of the proposed methodology due to the limited use of ecological and eco-mathematical modelling in the study. A concise mathematical representation of the model, which reflects the studied relationships in the data and may be derived from an econometric analysis, would be greatly appreciated.

Response #4.3:

We fully agree that the methodology needs to be clearly identified and presented. We have revised the paper accordingly, notably the methods section and the supplementary information. Essentially, the empirical work presented in the paper seeks to draw inferences from the data we have compiled on observable AQM activity. That is, we seek to identify how much AQM activity there is and whether its spatial distribution follows any generalizable patterns (with a focus on variation in economic and political conditions). One could call this approach Data Modeling. However, we do not fully understand what the reviewer means by eco-mathematical modeling. Essentially, we are using statistical methods (which means a form of applied mathematics) to better understand a particular form of environmentally relevant behaviour (monitoring activity). We found the following definition of eco-mathematical modeling: "Eco-mathematical modelling (also called ecological mathematical modelling) is the use of mathematics to represent, analyze, and predict ecological systems and processes. It combines ecology (the study of organisms and their environment) with mathematics (especially equations, statistics, and computational tools) to understand how living systems behave over time and space." As we are studying human/societal choices with respect to measuring air pollution we do not fully understand whether and why we should or could use what form of eco-mathematical modeling to analyze our data. In any event, our study is empirical, in the sense of trying to identify particular patterns in existing data, whereas computational modeling (e.g. agent based models) is simulating a particular system, with empirical data merely used to inform or calibrate a model. However, if the reviewer has specific suggestions on what we could do to assess the robustness of our main findings based on a particular alternative estimation strategy we would be happy to do so. Yet, we would need to know what this alternative data analysis strategy could be and why this might be as useful, or perhaps even better, for analyzing the particular type of data we are focusing on.

Reviewer #5:
Introduction

[Paragraph 3] You bring up applications of remote sensing and challenges with that, but the discussion needs to be more fleshed out. As currently written, it reads somewhat dismissive of the utility of remote sensing technology. There is a growing momentum and increasing acceptance for what is being termed as 'Air Quality Monitoring 2.0', where multiple sources (Legacy AQM, Remote Sensing, Low-cost sensors) are used in a hybrid way to get high-resolution data. There is a lot more acceptance of such monitoring even in the governments, e.g., India. However, even this approach does not eliminate the need for legacy AQM, which is the focus of this paper. Perhaps a better place for a more fleshed-out version of this paragraph could be in the discussion section.

Addition: I see that in the discussion section you have discussed this issue. Perhaps, a slight modification in language here would be sufficient. And useful to include a reference that reviews this new generation of air quality monitoring e.g. https://link.springer.com/article/10.1007/s41810-024-00281-1

Response #5.1:

Thank you for pointing out that our wording in the introduction could sound dismissive of remote sensing. This is not at all what we meant to say. We revised the introduction and discussion sections to highlight the complementarity of both on-the-ground AQM and remote-sensing technologies. We also revised the discussion section to flag this complementarity. For instance, in the introduction we now state:

"It is worth noting in this context that remote sensing data on air quality are becoming more widely available, e.g. for fine particulate matter (PM2.5). However, their spatial and temporal resolution is usually too coarse to support the design and implementation of well-targeted local clean air policies.[15–18] Recent discussions suggest,

however, that AQM networks should incorporate both remote and on-the-ground monitoring (AQM), to make the most of available data sources ([Kumari et al. 2025]; [Bagkis et al. 2025])."

In the discussion, we also added the following:

"Remote sensing is gaining in importance, with governments blending satellite data, legacy reference monitors, and low-cost sensor data. Yet, reference-grade monitoring remains essential for calibration and enforcement.

We also added a citation to recent research suggesting new directions for integrated AQM networks in the following sentence: "The findings presented here could encourage public authorities, international institutions, and civil society stakeholders to invest far more than hitherto the case into AQM, particularly in under-monitored poorer, and less democratic settings, drawing on lessons learned from [Bagkis et al. 2025] and [Anggraini et al. 2024]"

[Paragraph 8] Your choice of focusing on urban areas for this study needs to be justified better in the text. You could use supporting arguments like increasing population living in cities worldwide of LMICs, cities are where more population is exposed to poor air quality. When national governments start AQM, they often start with major city/urban areas and then expand; there are a few exceptions, though, where the beginning is near an industrial area away from the city, e.g., certain mining towns in some African countries. You can justify this choice better with relevant citations.

Response #5.2:

Thanks a lot for this comment. We agree that we should better justify the case selection, essentially the focus on cities as the unit of analysis, rather than countries. We added the following text to the introduction: "These problems are amplified by the fact that urban air pollution coincides and interacts with climate change and other ecological stressors, which implies that substantial synergies and co-benefits could be achieved from pursuing both clean air and climate mitigation measures (Cao et al. 2025, Dong et al. 2022; Vandyck et al. 2018). These challenges call for more air quality monitoring (AQM) to identify hotspots and design, implement, and evaluate clean air policy interventions. "

Data and Methods

[Paragraph 1] In 'Unit of Analysis' you write "the coordinates of so-called Urban Centres (UC).". Use of 'so-called' here reads very informal. You can replace it with something like "the coordinated of the Urban Centers (UC), as per the OECD definition"; I am assuming that's the source for you based on Ref #43

[Paragraph 2] Or perhaps you are not using the OECD definition? Are you using the OECD definition and applying it to non-OECD countries? Won't this be problematic? Have you made any adjustments? This part could use more clarity in your choice of definition of 'Urban Centres'

Also, this justification of focusing on non-OECD countries is better placed in Introduction section? A bit confusing – is it non-OECD or LMICs (Low-to-upper-middle-income). Are those turn out to be the exact same set of countries?

Response #5.3:

Thanks for encouraging us to clarify this. We clarified the language and source of the definition for the Urban Centres (UCs).

We are using the JRC-OECD definition as noted in [here], which defines an UC as a contiguous area with a minimum population threshold. Although this methodology was initially developed for cities in OECD countries, ref#43 provides a global dataset covering all countries. This is to our knowledge the best globally standardized definition of city units of various sizes. Although the citation for that methodology varies, this definition and GHS datasets have been used in various large scale environment studies such as https://www.sciencedirect.com/science/article/pii/S2542519622002601?via%3Dihub#bib20

https://www.sciencedirect.com/science/article/pii/S2542519621002552?via%3Dihub#bib45

https://www.pnas.org/doi/full/10.1073/pnas.2024792118#sec-2

https://www.sciencedirect.com/science/article/pii/S1618866722001819?via%3Dihub#sec0010

https://www.nature.com/articles/s43247-025-02270-9#Sec7

We edited the citation to point to the data product: https://human-settlement.emergency.copernicus.eu/ghs_smod2023.php

Some high-income countries are not part of the OECD, but all OECD countries are considered high-income. Thus, we edited our criteria of exclusion to only mention income based on the World Bank Classification from the World Urbanization Prospects 2018 dataset, as reported in the UC dataset.

[Paragraph 4] In 'Monitoring Stations' you write "With UCs as the unit of analysis, we merge data on reported terrestrial AQM activity from three different sources with global coverage, monitoring any activity up to mid-April 2024." Does it mean any AQM data is available up until mid-April 2024? Is there a threshold for duration? Do you expect AQM data to be current?

Response #5.4:

We include any AQM activity reported before mid-April 2024. We added a paragraph in the methods section that provides more information on the filtering "As an additional precaution, we exclude monitors that were reporting for less than 30 days, and those that reported for less than 2 weeks on the WAQI API in April 2024."

[Paragraph 5] There are a few countries in Africa where primarily low-cost sensor networks make up the AQM that are financially supported by international philanthropies working towards increasing air quality monitoring. While in some places/cities such projects have formal or informal blessing of governments, in many cases they don't. Would you not consider that exogenous as well? If not, why? One of your citations, Ref #27, focuses on AQM that are supported by the governments world-wide – a distinction like that could be helpful in your case?

Addition: I see that in the discussion section you have discussed this issue. It will still be worth noting in the methods that when you test a model where you exclude all low-cost sensors, the assumption is that low-cost sensors are not government-operated.

Response #5.5:

Thank you very much for these helpful comments. The data sources from which we derive our data on AQM activity does not include information on whether a sensor was installed and operated by a governmental or non-governmental entity, and by whom exactly. We tried to identify whether low-cost sensors were government or privately-owned, based on our interpretation of the name of the sensor, but eventually gave up.. E.g. "LRAPA-Emerald Park Inside" could be a likely example of a sensor setup by an official administration body, but provides little information whether it was set up by the local government, or an exogenous body. This means that reliably identifying whether an AQM activity is set up and run primarily by governmental or non-governmental actors, and by whom exactly, would require a major amount of additional research, probably based on case-by-case inquiries on site. This would be far beyond the scope of this paper. We edited the methods section to make it clearer that low-cost sensors may, or may not be government operated. "Unfortunately, these datasets do not always add enough information about the monitors, to assess whether they are governmentally or privately, or NGO operated. We expect most reference-grade monitors to be installed with the approval of the local or national government, while low-cost sensors could be set-up by any actor, including governmental entities."

[Paragraph 7] In 'Economic Resources', you write "Given that none of these UC has an observable monitoring station, we opt to exclude these 314 entities." Firstly, typo – UCs*. Secondly, did you consider any alternative approach instead of excluding these 314 entities? Perhaps, looking at nearby pixels and averaging incomes for surrounding pixels?

Response #5.6:

Thank you for pointing this out. Rather than extrapolating data from neighboring cities, we tested the robustness of the results using a different GDPpc dataset ([Kummu et al. 2025](#)) and got very similar results, see Supplementary Table 4. We added a section in the Supporting Information, titled "Robustness to a different income product" to describe how we incorporated this gridded dataset.

[Paragraph 9] In 'Air Pollution', you describe your choice of dataset, which is remote sensing-based but calibrated to the ground measurements. I believe this is probably the best available choice you had. But won't the accuracy of these measurements be biased against regions where there is no AQM? Important to mention this bias.

Response #5.7:

Thanks for noting this. We clarified this in the revised paper. Indeed, ground-level pollution estimates from remote sensing are more uncertain in places that do not report ground air pollution measurements, though they do not exhibit any systemic biases in the measurement. We revised the according section of the paper as follows: "These remote-sensed estimates of $PM_{2.5}$ *are built from models that rely on ground measurements, and thus carry more uncertainty in regions that do not have terrestrial AQM. The uncertainty in remotely sensed pollution should not lead to a systemic bias for cities that do not report on-the-ground measurements. (vanDonkelaar et al. 2016)"*

[Paragraph 10] In 'Conflict', you write "in over 1000 battle-related fatalities since its inception." Is this threshold based on similar work elsewhere?

Response #5.8:

We used this threshold based on the definition used in the UCDP codebook, using the cumulative_intensity variable described as follows: "This variable takes into account the temporal dimension of the conflict. It is a dummy variable that codes whether the conflict since the onset has exceeded 1,000 battle-related deaths. For conflicts with a history prior to 1946, it does not take into account the fatalities incurred in preceding years. A conflict is coded as 0 as long as it has not over time resulted in more than 1,000 battle-related deaths. Once a conflict reaches this threshold, it is coded as 1." see https://ucdp.uu.se/downloads/ucdpprio/ucdp-prio-acd-231.pdf

[Paragraph 12] In 'Other control variables': Why not use fixed effects for all countries? Why only India and China? You mention the third country Ethiopia also has a significant number of UC-AQM matches?

Response #5.9:

Thanks a lot for noting this and encouraging us to further reflect on this aspect of the paper. We choose not to add country-fixed effects because we are interested in both country and city level variables, rather than essentially filter out most cross-country differences via fixed effects. We pay special attention to China and India in the second paragraph of the result section: "China and India account for more than 2'100 and 380 monitors respectively, and over 1'800 and 3'100 cities respectively, with some cities among the most polluted globally (Fig. 2). This high concentration of AQM in China and India, and the high share of reference-grade (as opposed to low-cost sensor) monitoring in these countries requires special attention when exploring drivers of variation in AQM (see Data and Methods). However, it is also worth noting that China and India only rank 18th and 30th when normalizing the amount of AQM by urban population (Fig. 2). Figure 2 also shows that a large share of AQM in China and India takes place in cities experiencing medium to high levels of air pollution (remotely sensed). A much larger share of AQM in cities of other countries focuses on areas with relatively low to medium pollution levels (again remotely sensed). Again, this implies that we need to pay special attention to China and India when exploring the drivers of AQM, most notably how political systems and problem pressure act in combination."

Essentially, because India and China host many more cities than the rest of countries, ignoring this by simply using country fixed effects across the board would entail running a 'global-scale' analysis, where India and China carry most of the variation. To account for this, we downsample these two countries by randomly selecting n cities in each country, n being equal to the number of cities of the third country with the most cities, Ethiopia. The main results are robust to including all these cities, when including India and China fixed effects (see Figure 6). Also, we run a country-level poisson regression, in which results are consistent with the main findings. Our results also show that India and China differ from the rest of the cities of LMIC. We now added a paragraph discussing this finding and calling for future research on these two specific countries.

"Further, our results indicate that India and China diverge notably from other LMIC cities in their AQM coverage, reflecting different histories regarding air pollution management. China's nationwide AQM campaign led to a dense monitoring network (Liu et al., 2021) compared to other less-democratic countries experiencing high pollution. In contrast, at our cut-off in April 2024, India's monitoring infrastructure remained highly concentrated in Delhi (Gulia et al., 2022), making other cities in India less likely to have AQM compared to other polluted cities in democratic settings. Future research is needed to better describe why these countries behave as outliers compared to other polluted cities in democratic settings. Previous research in these countries are primarily descriptive, historical, or seek to identify whether more monitoring coincides or is followed by reduced air pollution. They do not systematically explain variation in AQM across cities, for

instance as a function of income levels and exogenously measured pollution. However, they could serve as a foundation for such work, though across political systems cannot be studied within countries. More research is needed to better understand why these countries behave as outliers compared to the other LMIC when looking at the role of political systems."

Finally, because Ethiopia has 500 cities, and the 4th country with most cities is Nigeria with 480, downsampling Ethiopian cities would not provide a sensible robustness test. Instead, we added a dummy for Ethiopia in our baseline model and plot the interaction for our main finding, see Figure Revision 1 below:

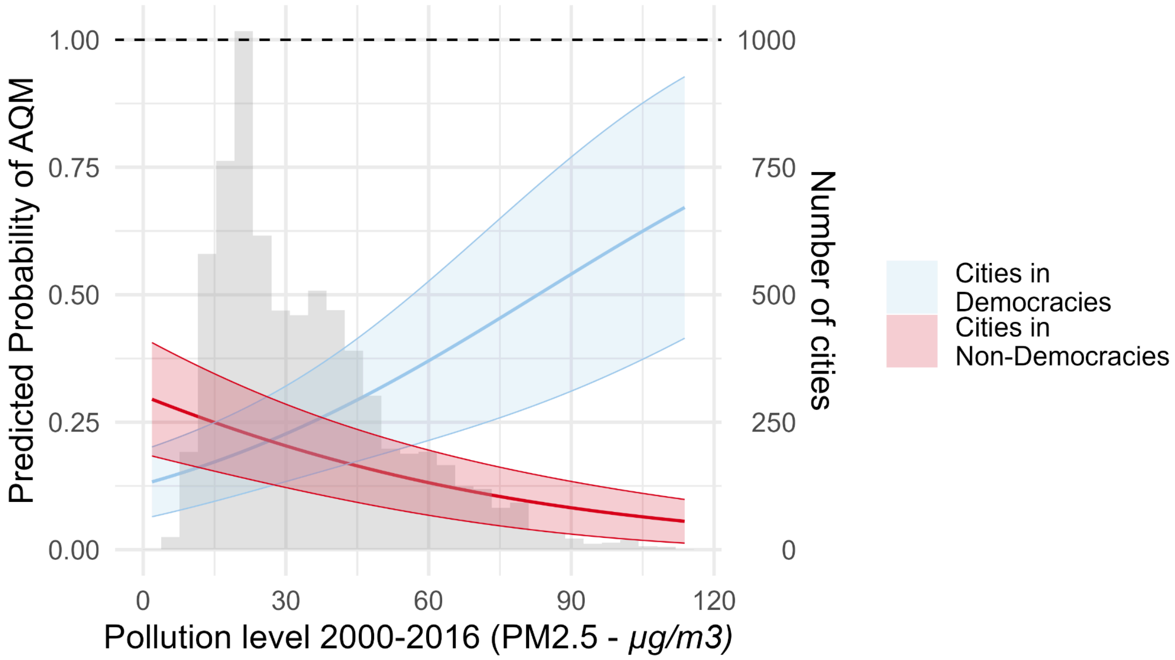

Figure Revision 1: Robustness check running our baseline model including a dummy for Ethiopia.

[Paragraph 13] In 'Data Analysis', it will be useful to provide equations for both multivariate analysis models.

Response #5.10:

Thanks for noting, we now include both equations.

[Paragraph 14] In 'Data Analysis', you write "To assess the robustness of the results, we use different combinations of decisions throughout the analysis and test them using multiverse analysis.". Replace 'decisions' with 'assumptions'?

Response #5.11:

Thanks for this comment, we have replaced decisions with assumptions.

Results

[Figure 1] It would be helpful if you also assigned different marker types with colours for different databases. It is a good practice to make the figures friendly to colour-blind readers and also advantageous if printed in black-and-white.

Response #5.12:

Thank you for pointing this out. Of course we want our Figure 1 to be as color-blind friendly as possible. It is difficult to represent 7 categories on a map in a readable way for color-blind people. After using software to simulate how our figure would be perceived by different color-blindnesses, we decided to use 4 different marker shapes. Each data source is displayed using a different shape, and any AQM location that overlaps in two or all data sources is displayed as a circle marker. We also edited the caption to explain this new design.

[Paragraph 3] You write "Less polluted cities are more likely to be monitored, and cities in nondemocratic settings tend to be more polluted ($p < 0.01$ & $p < 0.01$, permutation tests)." This could also be interpreted as the cities have lower pollution because they were monitored.

Response #5.13:

Thanks a lot for pointing this out. To isolate the potential impact of AQM on pollution level, we tested whether using pollution data from 2000-2008 changes the described effect, which is not the case ($p < 0.01$, permutation test, difference = 4.57). We edited the previously ambiguous wording: "Cities with lower pollution levels are more likely to have air quality monitoring ($p < 0.01$, permutation test, difference = 3.93). Comparing political contexts, cities in non-democratic settings tend to be more polluted than those in democratic settings ($p < 0.01$, permutation test, difference = 8.25). However, within both regime types, cities that have air quality monitoring (AQM) are significantly less polluted on average, with the difference being larger for democratic cities ($p < 0.01$ for both, permutation tests; differences of 2.01 and 4.97, respectively)."

[Figure 4] So the effect sizes are in numerical values? The dependent variable is the Presence of AQM, correct? What is the threshold for the classification in this logistic regression? It'd be useful to connect caption to your multivariate regression equation provided earlier.

Response #5.14:

The effect size is in numerical value, with a value closer to 1 indicating a strong likelihood for a city to host a monitor. We made this clearer in the figure caption. Because most cities in LMICs do not host a monitor, the mean predicted values are rather closer to 0. In a balanced dataset with 50% cities hosting a monitor, mean predicted probability would be closer to 0.5 and could be used as a threshold (though we do not use our model for predictions, and do not need to make any assumptions). We added a link to equation 1 in the caption of the figure.

[Paragraph 8] You write "Although the effect is weaker for reference-grade monitors, it remains statistically significant when applying what we consider the most appropriate estimation strategy (Fig. 6)." I am assuming you are referring to the combination of 'Everything – embassies' with 'IND/CHN subsampled'. Calling it 'best estimation strategy' is misleading. You can refer to it as your baseline model – or something to

that order – and refer it like that throughout the paper. Earlier, when you mentioned decisions, you can refer to it as the baseline model again.

Response #5.15:

Thanks for noting this. We changed the wording throughout the paper, using 'baseline model' instead.

Discussion

Your special treatment of India and China, justifiably so due to the high number of urban centers and more AQM data, makes me curious about AQM journey of these two countries. A simple search on Google Scholar results in several studies that examine AQM status/drivers/history for these two countries. Perhaps synthesizing some of them and including a brief discussion of how those findings may align with your findings will enrich this discussion. Quantitative analysis that relies on proxies of complex socioeconomic parameters (e.g., democracy) to find causal mechanisms of policies (here, AQM) gets more grounding when compared to findings from qualitative research.

The following citations may be useful in this case:

https://doi.org/10.1016/j.jenvman.2019.110031 - history of monitoring in China

https://doi.org/10.1016/j.jenvman.2021.113232 - an analysis similar to yours focussed on cities in China

https://doi.org/10.1016/j.envc.2021.100431 - history of monitoring in India

Response #5.16:

Thanks a lot for pointing us to this. We have flagged this as an important issue for further research in the discussion section, see response#5.9. There are two particularly noteworthy studies on AQM in India and China respectively (Liu et al. 2021; Gulia et al. 2022). Both of these are primarily descriptive or historical, or seek to identify whether more monitoring coincides or is followed by reduced air pollution. They do not seek to systematically identify the determinants of variation in AQM across cities, for instance as a function of income levels and exogenously measured pollution. However, they could serve as a foundation for such an analysis, the limitation that differences across political system types cannot be studied within countries, where the political system remains constant.

Comments from the editor:

Please state what role the funders took in the study. If the funders had no role, please state: "The funders had no role in study design, data collection and analysis, decision to publish, or preparation of the manuscript."

If this statement is not correct you must amend it as needed.

Please include this amended Role of Funder statement in your cover letter; we will change the online submission form on your behalf.

Response #E.1:

We inserted this statement: The funders had no role in study design, data collection and analysis, decision to publish, or preparation of the manuscript.

5. We note that Figure 1 in your submission contain [map/satellite] images which may be copyrighted. [...] We require you to either (1) present written permission from the copyright holder to publish these figures specifically under the CC BY 4.0 license, or (2) remove the figures from your submission. [...]

Response #E.2:

Figure 1 does not include images that are copyrighted. Figure 1 was produced with our original code, relying on the AQM dataset described in Methods, and the shapefile for countries from Natural Earth. We added a citation to the Natural Earth package in the Figure description. No permission is needed to use Natural Earth. Crediting the authors is unnecessary. So, from our viewpoint, there is no problem with copyrights in this case.
