# Supplementary material for: Large gaps in monitoring urban air pollution in low- and middle- income countries associated with economic conditions and political institutions
Source: PLoS One. 2026 Jan 13;21(1):e0321358. doi: 10.1371/journal.pone.0321358 (PMC12798989; doi:10.1371/journal.pone.0321358)
Supplement: Attachment — Submitted filename: PONE-D-25-11715_reviewer - Comments.docx [file pone.0321358.s002.docx]

Introduction

[Paragraph 3] You bring up applications of remote sensing and challenges with that, but the discussion needs to be more fleshed out. As currently written, it reads somewhat dismissive of the utility of remote sensing technology. There is a growing momentum and increasing acceptance for what is being termed as ‘Air Quality Monitoring 2.0’, where multiple sources (Legacy AQM, Remote Sensing, Low-cost sensors) are used in a hybrid way to get high-resolution data. There is a lot more acceptance of such monitoring even in the governments, e.g., India. However, even this approach does not eliminate the need for legacy AQM, which is the focus of this paper. Perhaps a better place for a more fleshed-out version of this paragraph could be in the discussion section.
*Addition: I see that in the discussion section you have discussed this issue. Perhaps, a slight modification in language here would be sufficient. And useful to include a reference that reviews this new generation of air quality monitoring e.g.* [*https://link.springer.com/article/10.1007/s41810-024-00281-1*](https://link.springer.com/article/10.1007/s41810-024-00281-1)

[Paragraph 8] Your choice of focusing on urban areas for this study needs to be justified better in the text. You could use supporting arguments like increasing population living in cities worldwide of LMICs, cities are where more population is exposed to poor air quality. When national governments start AQM, they often start with major city/urban areas and then expand; there are a few exceptions, though, where the beginning is near an industrial area away from the city, e.g., certain mining towns in some African countries. You can justify this choice better with relevant citations.

Data and Methods

[Paragraph 1] In ‘Unit of Analysis’ you write *“the coordinates of so-called Urban Centres (UC).”*. Use of ‘so-called’ here reads very informal. You can replace it with something like “*the coordinated of the Urban Centers (UC), as per the OECD definition”*; I am assuming that’s the source for you based on Ref #43

[Paragraph 2] Or perhaps you are not using the OECD definition? Are you using the OECD definition and applying it to non-OECD countries? Won’t this be problematic? Have you made any adjustments? This part could use more clarity in your choice of definition of ‘Urban Centres’

Also, this justification of focusing on non-OECD countries is better placed in Introduction section? A bit confusing – is it non-OECD or LMICs (Low-to-upper-middle-income). Are those turn out to be the exact same set of countries?

[Paragraph 4] In ‘Monitoring Stations’ you write *“With UCs as the unit of analysis, we merge data on reported terrestrial AQM activity from three different sources with global coverage, monitoring any activity up to mid-April 2024.”* Does it mean any AQM data is available up until mid-April 2024? Is there a threshold for duration? Do you expect AQM data to be current?

[Paragraph 5] There are a few countries in Africa where primarily low-cost sensor networks make up the AQM that are financially supported by international philanthropies working towards increasing air quality monitoring. While in some places/cities such projects have formal or informal blessing of governments, in many cases they don’t. Would you not consider that exogenous as well? If not, why? One of your citations, Ref #27, focuses on AQM that are supported by the governments world-wide – a distinction like that could be helpful in your case?
*Addition: I see that in the discussion section you have discussed this issue. It will still be worth noting in the methods that when you test a model where you exclude all low-cost sensors, the assumption is that low-cost sensors are not government-operated.*

[Paragraph 7] In ‘Economic Resources’, you write *“Given that none of these UC has an observable monitoring station, we opt to exclude these 314 entities.”* Firstly, typo – UCs*. Secondly, did you consider any alternative approach instead of excluding these 314 entities? Perhaps, looking at nearby pixels and averaging incomes for surrounding pixels?

[Paragraph 9] In ‘Air Pollution’, you describe your choice of dataset, which is remote sensing-based but calibrated to the ground measurements. I believe this is probably the best available choice you had. But won’t the accuracy of these measurements be biased against regions where there is no AQM? Important to mention this bias.

[Paragraph 10] In ‘Conflict’, you write “in over 1000 battle-related fatalities since its inception.” Is this threshold based on similar work elsewhere?

[Paragraph 12] In ‘Other control variables’: Why not use fixed effects for all countries? Why only India and China? You mention the third country Ethiopia also has a significant number of UC-AQM matches?

[Paragraph 13] In ‘Data Analysis’, it will be useful to provide equations for both multivariate analysis models.

[Paragraph 14] In ‘Data Analysis’, you write “To assess the robustness of the results, we use different combinations of decisions throughout the analysis and test them using multiverse analysis.”. Replace ‘decisions’ with ‘assumptions’?

Results

[Figure 1] It would be helpful if you also assigned different marker types with colours for different databases. It is a good practice to make the figures friendly to colour-blind readers and also advantageous if printed in black-and-white.

[Paragraph 3] You write “Less polluted cities are more likely to be monitored, and cities in nondemocratic settings tend to be more polluted (p < 0.01 & p < 0.01, permutation tests).” This could also be interpreted as the cities have lower pollution because they were monitored.

[Figure 4] So the effect sizes are in numerical values? The dependent variable is the Presence of AQM, correct? What is the threshold for the classification in this logistic regression? It’d be useful to connect caption to your multivariate regression equation provided earlier.

[Paragraph 8] You write “Although the effect is weaker for reference-grade monitors, it remains statistically significant when applying what we consider the most appropriate estimation strategy (Fig. 6).” I am assuming you are referring to the combination of ‘Everything – embassies’ with ‘IND/CHN subsampled’. Calling it ‘best estimation strategy’ is misleading. You can refer to it as your baseline model – or something to that order – and refer it like that throughout the paper. Earlier, when you mentioned decisions, you can refer to it as the baseline model again.

Discussion

Your special treatment of India and China, justifiably so due to the high number of urban centers and more AQM data, makes me curious about AQM journey of these two countries. A simple search on Google Scholar results in several studies that examine AQM status/drivers/history for these two countries. Perhaps synthesizing some of them and including a brief discussion of how those findings may align with your findings will enrich this discussion. Quantitative analysis that relies on proxies of complex socioeconomic parameters (e.g., democracy) to find causal mechanisms of policies (here, AQM) gets more grounding when compared to findings from qualitative research.

The following citations may be useful in this case:

<https://doi.org/10.1016/j.jenvman.2019.110031> - history of monitoring in China

<https://doi.org/10.1016/j.jenvman.2021.113232> - an analysis similar to yours focussed on cities in China

<https://doi.org/10.1016/j.envc.2021.100431> - history of monitoring in India
